# The Book World of Medicine and Science

**Published:** 1896-04-18

**Authors:** 


### THE BOOK WORLD OF MEDICINE AND SCIENCE.

The Art of Compounding. By W. L. Scoville. (London: Kegan Paul, Trench, Trübner, and Co. 1896. Price 12s. 6d.)

Mr. Scoville is Professor of Applied Pharmacy in the laboratory of the Massachusetts College of Pharmacy, and in that capacity has had considerable experience in teaching. Such a qualification makes him fully competent to write a book of this description, and by enlarging its scope he has endeavoured to make his work of value not only to students but to dispensers who are already engaged in practice. We can confidently recommend the present volume as a handy reference book at the prescription counter, as in it will be found collected directions for prescribing those mixtures which frequently are a source of confusion when required to be dispensed in a hurry, and for compounding new drugs included by the physician in what is otherwise a well-known prescription. American pharmacy after all is so little different from our own that the fact of this book appealing primarily to dispensers working under the American Pharmacopœia need not be any hindrance to those who feel that they would like to supplement their knowledge of dispensing in this country. Some of the phases are at times strange to English readers, as for example fluidram and fluidrachm, which are used apparently by the author as equivalents. The chapters devoted to the compounding of pills and emulsions are especially well written, and contain a large amount of information which should lessen considerably the difficulties which beset the beginner in compounding these preparations.

Premature Burial. London: Swan Sonnenschein and Co. (Limited), Paternoster Square, E.C.

The title of this book is used as a peg on which to hang some of the author's pet theories on the human soul. There is no objection to that, so long as the reader understands the true inwardness of the situation. On the question of premature burials there need be no doubt entertained as to the fact. Men and women, probably are sometimes buried alive. Some of them are unconscious at the time of interment, and never recover consciousness. These suffer nothing. Others are buried whilst yet either unconscious, or conscious and destitute of the power to make known their condition. These may even know what is happening at the very time of their burial, and sometimes after they are hopelessly immured in their dreadful prison house, may regain the power of speech and movement, and die enduring agonies untold. For the sake of such it is well for medical men—and all human beings beside—to remember that the only really infallible sign of death is the unmistakeable presence of commencing putrefaction. With regard to Dr. Hartmann's theories of the separability of the soul from the body, and the reunion of the two whilst life is still in progress, we can only advise readers to read for themselves. Those who love horrors will find them in plenty; writers of short stories may unearth one or two plots which have not previously being "borrowed"; and students of transcendental psychology will not improbably enjoy the author's speculations in an ancient, but still facinating field. The price of the little book is a shilling, and in our judgment it is well worth the money.

Injuries and Diseases of the Genital and Urinary Organs. By Henry Morris, M.A., M.B.Lond., F.R.C.S. (Cassell and Co., Limited, London, Paris, and Melbourne. 1895, pp. 494. Illustrated.)

Mr. Henry Morris has now completed his work on the urinary organs, as this volume must be looked upon as an addition to his book, entitled "Surgical Diseases of the Kidneys." This recent work is, without doubt, one of the best on this important subject, and is full of sound principles and useful hints both to general practitioners and students. We should like to see in any future edition more details concerning some of the operations mentioned, and we think it would have been more valuable as a work of reference if the author had gone a little more deeply into the treatment of some of the conditions so ably described and illustrated. We can strongly recommend the work, and we look upon it as one of the standard books on the subject.

Handbook of the Diagnosis and Treatment of Skin Diseases By Arthur Van Harlingen, M.D., Emeritus Professor of Dermatology in the Philadelphia Polyclinic. (Philadelphia: P. Blakiston, Son, and Co. London: Kegan Paul, Trench, Trübner, and Co. 1895. Price 12s.)

In writing this book the author says that he has had in his mind the wants of the practitioner, and that he has therefore given space to the description, diagnosis, and treatment of the various affections, while touching lightly on questions of etiology, and omitting for the most part all reference to pathological anatomy. There is, however, a further reason why this must be looked upon as a practitioner's rather than a student's book, and that is that its arrangement is such—being purely alphabetical—that until the reader is sufficiently familiar with the various diseases of the skin to be able to give each of them a name, the book is practically closed to him. This is a pity, for there is a good deal in it that is very useful. A great feature is made of differential diagnosis, the points of distinction between the various diseases which are likely to be mistaken for each other being given in parallel columns. The directions for treatment, which are common-sense and practical, are culled from many sources, in addition to being the outcome of what is evidently a large personal experience. In most cases the necessary prescriptions are given in the text, a plan which offers considerable advantages to the reader. The work is well illustrated so far as the woodcuts go, but the few chromo-lithographs which are given are very poor indeed, and, in fact, are quite useless. It should be added that there is no index, and that the cross references are by no means perfect. These are great blemishes, and much limit the utility of a book which all the same will prove a useful and suggestive guide to those who do not care for a consecutive account of disease of the skin, but merely wish to refer to what is known concerning any particular malady.

